# A voluntary deductible in health insurance: the more years you opt for it, the lower your premium?

**DOI:** 10.1007/s10198-016-0767-4

**Published:** 2016-02-09

**Authors:** K. P. M. van Winssen, R. C. van Kleef, W. P. M. M. van de Ven

**Affiliations:** Institute of Health Policy and Management, Erasmus University Rotterdam, Burgemeester Oudlaan 50, 3062 PA Rotterdam, The Netherlands

**Keywords:** Adverse selection, Health insurance, Moral hazard, Premium, Voluntary deductibles, D82, G22, I11, I13

## Abstract

Adverse selection regarding a voluntary deductible (VD) in health insurance implies that insured only opt for a VD if they expect no (or few) healthcare expenses. This paper investigates two potential strategies to reduce adverse selection: (1) differentiating the premium to the duration of the contract for which the VD holds (ex-ante approach) and (2) differentiating the premium to the number of years for which insured have opted for a VD (ex-post approach). It can be hypothesized that premiums will decrease with the duration of the contract or the number of years for which insured have opted for a VD, providing an incentive to insured to opt for a deductible also in (incidental) years they expect relatively high expenses. To test this hypothesis, we examine which premium patterns would occur under these strategies using data on healthcare expenses and risk characteristics of over 750,000 insured from 6 years. Our results show that, under the assumptions made, only without risk equalization the premiums could decrease with the duration of the contract or the number of years for which insured have opted for a VD. With (sophisticated) risk equalization, decreasing premiums seem unfeasible, both under the ex-ante and ex-post approach. Given these findings, we are sceptical about the feasibility of these strategies to counteract adverse selection.

## Introduction

In several regulated health insurance markets, such as Germany, Switzerland, the Netherlands, and the US, insured are offered the possibility to opt for a voluntary deductible in return for a premium rebate. These deductibles may counteract moral hazard [10, 43], which is a well-known consequence of (comprehensive) health insurance and refers to the change in health behavior and healthcare consumption caused by the fact that the insurer reimburses (part of) the costs. Economic theory predicts that rational consumer behavior causes individuals to opt for a voluntary deductible only if the expected expenses under the deductible fall below the premium rebate. This phenomenon is referred to as adverse selection and implies that low-risk individuals are more inclined to opt for a voluntary deductible than high-risk individuals within the same premium-risk group [1, 7, 9, 24, 25]. Such behavior would also imply that insured do not opt for a deductible in a (incidental) year they expect (high) expenses. This may limit the moral hazard reduction resulting from the deductible.

In free markets, insurers can reduce adverse selection by risk-rating the premium or by denying insured to reduce the deductible level (or metal tier) in later years. Furthermore, insurers in either free or regulated markets can reduce adverse selection by an ex-ante or ex-post differentiation of the premium according to, respectively, the duration of the contract for which the voluntary deductible holds or the number of previous years for which insured have opted for a voluntary deductible.^1^ It is hypothesized that the longer the period for which the voluntary deductible holds or the more previous years insured have opted for a voluntary deductible, the lower the premium can be. This could incentivize insured to opt for a voluntary deductible for a longer period or more consecutive years, implying a larger moral hazard reduction since insured then also opt for a voluntary deductible in (incidental) years they expect high expenses. Related to the ex-ante differentiation, the German law states that the deductible holds for at least 3 years. Related to the ex-post differentiation, the Dutch law offers insurers the possibility to differentiate the premium rebate to the number of years insured have opted for a voluntary deductible.

This paper explores the premium patterns in case of either an ex-ante or ex-post differentiation of the premium. Our central research question reads: What would the premiums look like when differentiated to either the duration of the contract for which the voluntary deductible holds (i.e., the ex-ante approach) or the number of previous consecutive years insured have opted for a voluntary deductible (i.e., the ex-post approach)? These premiums depend upon the predicted expenses of insured choosing the different deductible options. However, for which deductible option insured choose depends on their predicted expenses and the premium. Our variable of interest, the premium, therefore is an endogenous variable. Consequently, we have to simulate the distribution of insured across the deductible options and subsequently determine the corresponding premiums. To achieve this, we use data on healthcare expenses and risk characteristics of 762,982 insured from 6 years.

The “Theoretical background” discusses the moral hazard reduction resulting from deductibles, how to counteract adverse selection in regulated markets, the composition of the premium and the effect of risk equalization on the premium. The data and methods are explained in “Data” and “Methods”. Subsequently, the results, conclusions and discussion are presented in, respectively,  “Results”, “Conclusions” and “Discussion”.

## Theoretical background

### Moral hazard and deductibles

Moral hazard is a well-known consequence of comprehensive health insurance, such as in Germany, Switzerland, the Netherlands, and the US. It refers to the change in health behavior and healthcare consumption resulting from the reimbursement of the costs for healthcare services by the insurer. It could be counteracted by cost-sharing arrangements. Many have studied the effect of different cost-sharing arrangements on the moral hazard reduction [7, 10, 12, 13, 30, 44]. This paper focuses on the voluntary deductible as an instrument to reduce moral hazard. Gerfin et al. [13] show that due to high voluntary deductibles, healthcare demand in Switzerland dropped by 27 %. Additionally, Trottmann et al. [30] correct for the selection effect that results from the voluntary deductible and show that high voluntary deductibles in Switzerland reduced healthcare expenses by 23 %. These studies thus show that deductibles could indeed be an effective instrument to counteract moral hazard.

### Counteracting adverse selection in regulated health insurance markets

Rational economic behavior predicts that individuals will only opt for a voluntary deductible if their expected out-of-pocket expenses under the deductible are smaller than the offered premium rebate. This could lead to adverse selection, meaning that low-risk individuals are more inclined to opt for a voluntary deductible than high-risk individuals within the same premium-risk group [1, 24, 25]. Eventually, this could result in an adverse selection (or death) spiral. Several studies indicate that the key conditions for adverse selection—the ability to forecast risk and the fact that this forecast affects insurance takeout [6, 27, 40]—exist [19, 21, 22]. Insurers in free markets can reduce adverse selection by risk-rating the premium or by denying insured to reduce their deductible level in later years. However, insurers in regulated health insurance markets, such as in Germany, Switzerland, the Netherlands, and the US, do not have these options. In these markets, both the premium and the premium rebate for voluntary deductibles must be community-rated, meaning that insurers must offer the same premium (rebate) to each insured with the same insurance policy and the same deductible level.^2^ Additionally, the above-mentioned countries have open enrolment, which means that applicants cannot be rejected. Therefore, insured can determine each year whether to opt for a voluntary deductible.^3^ The requirements of both community-rating and open enrolment cause adverse selection to be larger in regulated markets than in free markets, which limits the moral hazard reduction resulting from voluntary deductibles. Insurers in regulated markets may have two options to reduce adverse selection.^4^ The first option regards an ex-ante differentiation of the premium to the duration of the contract for which the voluntary deductible holds (e.g., 1, 5, 10 years, etc.). The second option regards an ex-post differentiation of the premium to the number of previous consecutive years insured have opted for a voluntary deductible. Note that with this option, compared to the ex-ante option, insured have the possibility to adjust (or even opt out of) the deductible level each year. Assuming that insured who opt for a voluntary deductible for a longer contract period or in multiple consecutive years are healthier than insured who only opt for a voluntary deductible in 1 year, it can be hypothesized that the premium would, ceteris paribus, decrease with the contract period for which the deductible holds or the number of previous consecutive years insured have opted for a voluntary deductible. In Germany, the law states that voluntary deductibles hold for 3 years, which is related to the ex-ante differentiation as discussed within this paper, except that German insured have no choice regarding the contract period (i.e., insured either choose no deductible or a deductible that holds for 3 years). The Dutch law provides insurers the possibility to apply an ex-post differentiation of the premium as discussed within this paper, stating that “the premium rebate may depend on the number of calendar years for which the insured has opted for a voluntary deductible”.^5^ It was mentioned that insured had to weigh the increase in premium rebate against the possibility to decrease the voluntary deductible [15] and that this would provide insured with an incentive to opt for a deductible also in a year they incidentally expect (high) healthcare expenses. After a decade, however, none of the Dutch insurers utilizes the option.

### Composition of the premium

**Fig. 1:**
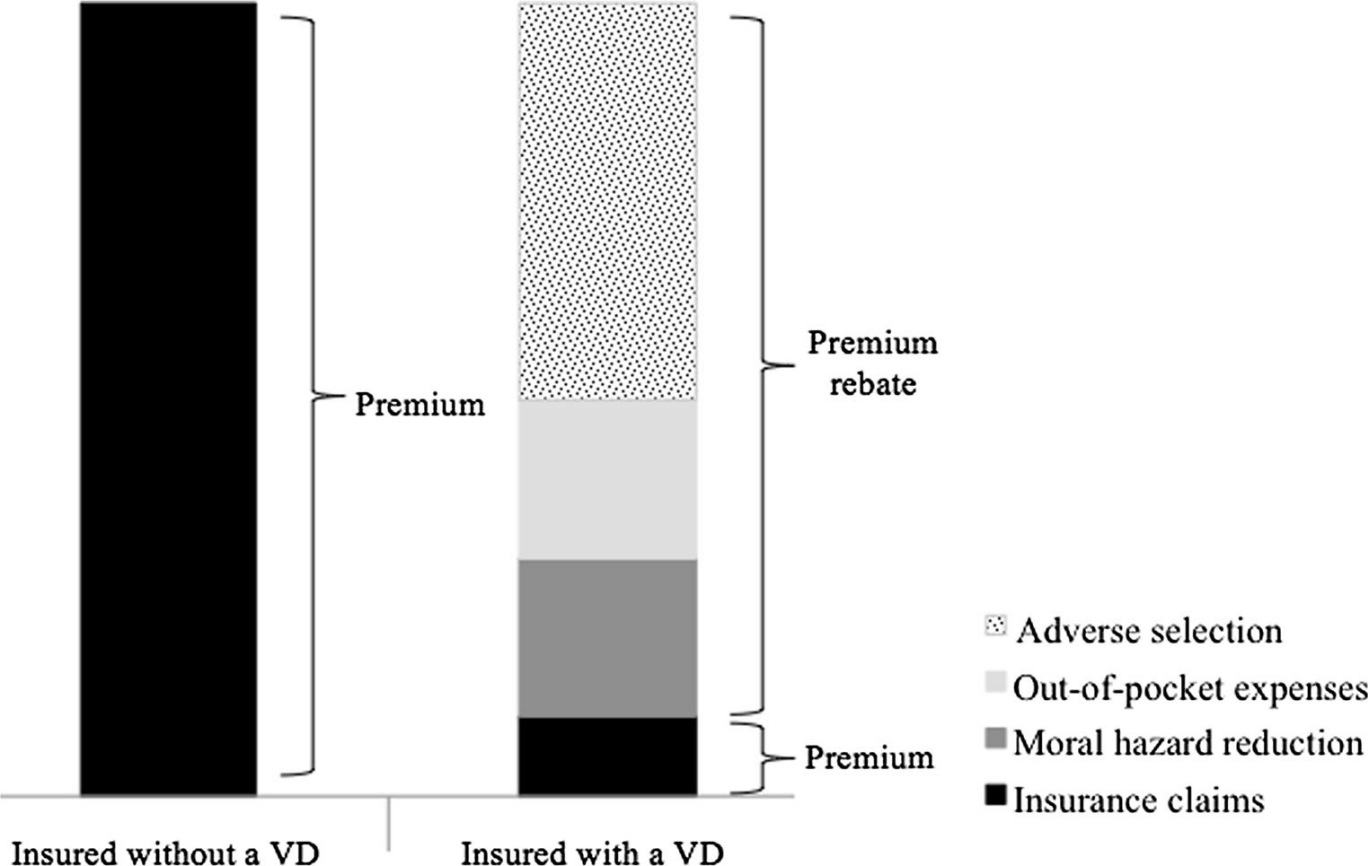
Composition of the premium and premium rebate in health insurance markets without risk equalization

To determine the ex-ante and ex-post differentiated premiums, this section discusses the composition of the premium. Generally speaking, the premium paid by insured equals the expected insurance claims^6^ (see Fig. 1). Insured with a voluntary deductible receive a premium rebate that can be decomposed into three components [33]. The first component is the effect of self-selection that arises because, given a certain premium rebate, healthy insured have a greater incentive to opt for a voluntary deductible than unhealthy insured (i.e., the adverse selection component). Consequently, market segmentation is created where insured with a voluntary deductible are on average healthier and have lower insurance claims than insured without a voluntary deductible. The second component is the moral hazard reduction resulting from the voluntary deductible, which (ceteris paribus) lowers the total healthcare expenses. The third component regards the expected out-of-pocket expenses paid by insured with a voluntary deductible. Consequently, the insurer has to reimburse less than for insured without a voluntary deductible (ceteris paribus).

Many studies show that mostly young and healthy insured opt for a voluntary deductible (e.g., [8, 11, 20, 31, 41]). Given these findings, it is expected that insured who opt for a voluntary deductible with a long contract period or in more consecutive years are healthier than insured with short contract periods and few years. Subsequently, the expected insurance claims, moral hazard reduction, and out-of-pocket expenses of insured with a voluntary deductible for a long contract period or in more consecutive years could be smaller than for insured with short contract periods or few years. So, if the premium is either ex-ante or ex-post differentiated, one possible outcome could be that the premium decreases with respectively the contract period or the number of years insured have opted for a voluntary deductible. However, insured with long contract periods could incur (unexpected) high healthcare expenses during the contract. As a result, the differentiation could also result in an increasing premium with the contract period. Furthermore, if the ex-post differentiation indeed results in a behavioral effect (i.e., insured keep the deductible also in years they expect high healthcare expenses), the premium could also increase with the number of years insured have opted for a deductible.

### Effect of risk equalization on the premium

In addition to the requirements of community-rating (i.e., insurers must offer the same premium to each insured with the same policy and the same deductible level) and open enrolment (i.e., applicants cannot be rejected), Germany, Switzerland, the Netherlands, and the US have a risk equalization scheme that compensates insurers for differences in predicted expenses between low-risk and high-risk individuals. The current German risk equalization scheme includes demographic risk adjusters and a set of morbidity-based risk adjusters [4]. The Swiss risk equalization scheme includes age, gender, and prior hospitalization as risk adjusters [28]. The Dutch scheme includes an age and gender interaction, Pharmacy-based Cost Groups (PCGs), Diagnoses-based Cost Groups (DCGs), durable medical equipment cost groups, source of income, region, social economic status, multiple-year high costs and generic somatic morbidity [16]. In 2014, the Health Insurance Marketplaces in all states in America (except Massachusetts) use the risk equalization model developed by the US Department of Health and Human Services based upon the Hierarchical Condition Categories [17]. These risk equalization systems affect the premium paid by insured. Let us assume that risk equalization perfectly adjusts for the differences in predicted expenses between low-risk and high-risk individuals. In that case, the premium consists of the expected insurance claims and a risk equalization payment. Firstly, for insured without a voluntary deductible, this risk equalization payment is equal to the difference between the average healthcare expenses in the population and the individuals’ average predicted insurance claims. This implies that risk equalization has a negative effect on the premium if the individual expenses are larger than the average expenses, and a positive effect on the premium if the individual expenses are smaller than the average expenses. Secondly, the risk equalization payment for insured with a voluntary deductible equals the difference between the average healthcare expenses in the population and the sum of the predicted expenses, the moral hazard reduction, and the out-of-pocket expenses resulting from the voluntary deductible. After all, risk equalization aims to equalize the adverse selection component. In case of perfect risk equalization, this adverse selection component is fully equalized and incorporated into the premium. Therefore, the premium rebate only consists of the moral hazard reduction and out-of-pocket expenses. However, if risk equalization does not perfectly adjust for differences in predicted expenses between low-risk and high-risk individuals, a share of the adverse selection component is not equalized and therefore reflected into the premium rebate. The difference in quality between the Swiss and the Dutch risk equalization schemes might also (partially) explain why in Switzerland 56 % of the insured opted for a voluntary deductible in 2013 [5], while in the Netherlands only 12 % opted for a voluntary deductible in 2015 [39]. After all, Swiss insurers might be able to reflect a larger share of the adverse selection component into the premium rebate.

### Conclusion

In sum, voluntary deductibles are offered in the regulated health insurance markets of Germany, Switzerland, the Netherlands, and the US in order to counteract moral hazard. However, the moral hazard reduction is limited due to adverse selection, where insured only opt for a voluntary deductible if their expected expenses are smaller than the premium rebate. In these regulated markets, adverse selection could potentially be reduced by an ex-ante or ex-post differentiation of the premium to respectively the contract period for which the voluntary deductible holds or the number of previous consecutive years an insured has opted for a voluntary deductible. To determine the differentiated premiums that could then be offered, we perform several empirical simulations in which we also include the effect of risk equalization on the premium.

## Data

For the empirical analyses, we use the Achmea Health Database that contains administrative data from a large Dutch health insurer who operates mainly in the western and eastern parts of the Netherlands. It includes individual-level information on insurance claims in the years 2006–2011 aggregated at and categorized into the following 11 types of healthcare services: GP-care, pharmacy, inpatient care, hospital admissions, outpatient care, dental care, maternity care, durable medical equipment, physiotherapy, mental healthcare, and care consumed in a foreign country. Moreover, the database includes an encrypted ID number and (per-year) information on the year of birth, sex, ethnicity, degree of urbanization, the number of days of enrolment, and in which PCG and/or DCG the insured is classified for the risk equalization scheme. Remember that PCGs and DCGs are risk adjusters used as a proxy for health status, based upon prior use of pharmaceuticals and prior hospital inpatient diagnoses, respectively [37]. For simplicity reasons, two selection criteria are applied to our simulation sample: individuals must be fully insured in all 6 years^7^ and individuals must be 18 years or older on January 1, 2007, since in the Netherlands only adults can opt for a voluntary deductible. These selection criteria provide us with a sample of 762,982 insured. In order to be able to compare the premium of both the ex-ante and ex-post differentiation and since the deductible amount remains the same for all years (see “Composition of the premium”), all healthcare expenses are corrected for inflation to the level of 2007. When comparing our sample with the Dutch population, it shows that the average health in the dataset is somewhat worse compared to the Dutch population: e.g., more insured are classified into a PCG or DCG and the average healthcare expenses are higher. This is probably caused by the fact that the Achmea Health Database belongs to a former sickness fund. Since we select insured opting for a voluntary deductible relative to the entire sample, the difference between our sample and the Dutch population could affect the absolute level of the premium but would not affect the premium patterns over the deductible options.

## Methods

### Opting for a voluntary deductible

In order to determine the premiums in year *t*, we need to know which insured opt for which deductible option. In other words, for the ex-ante differentiation, we need to know for year *t* which group of insured did not opt for a voluntary deductible and which groups of insured opted for a voluntary deductible with either a 1-, 2-, 3-, 4-, or 5-year contract period. For the ex-post differentiation, we need to know for year *t* which group of insured did not opt for a voluntary deductible, which group opted for a voluntary deductible in year *t*, but not in year *t* − 1, which group opted for a voluntary deductible in year *t* and *t* − 1, but not in year *t* − 2, etc.

Following the theory of rational consumer behavior, the distributions of insured over the deductible options would ideally be determined by comparing the insured’s expected benefits with his/her expected costs of opting for a voluntary deductible, implying that only insured for whom their expected healthcare expenses under the deductible are smaller than the premium rebate would opt for a voluntary deductible. However, whether insured opt for a voluntary deductible in a certain year depends on the premium, but at the same time the premium depends on the distribution of insured over the deductible options. This makes the premium, which is also our variable of interest, an endogenous variable. This means that we cannot use the premium as the input variable to determine who opts for a deductible. Therefore, to get an estimate of these premiums, we make assumptions about the distribution of insured over the deductible options for both the ex-ante and ex-post differentiation. We assume that insured with the lowest predicted healthcare expenses would opt for a deductible. Several models, which can be found in “Appendix 1”, to determine the rank of insured based upon their predicted healthcare expenses in year *t* have been tested. The most accurate model, based upon the Spearman’s correlation coefficient, is an OLS model with a log transformation of healthcare expenses and we use this model for our empirical simulations. The dependent variable regards the total healthcare expenses under basic insurance in year *t*. The independent variables indicate several background characteristics that are in the dataset: an age and gender interaction, classification into a PCG and/or DCG in year *t* (based upon information from year *t* − 1), degree of urbanization in the residential area, ethnicity and past total healthcare expenses in year *t* − 1 classified into vigintiles. A detailed description of the independent variables can be found in “Appendix 2”. After the healthcare expenses are predicted, insured are ranked accordingly. Furthermore, we determine the rank of insured in years *t* − 1, *t* − 2, *t* − 3, and *t* − 4 based upon their predicted healthcare expenses in those years. In order to do so, we use the same model specification as for year *t*, but the variables are based upon data from earlier years.^8^ Note that for the ex-ante differentiation, insured have to decide on the duration of their contract period in year *t* (i.e., 2007) and that we use information from the years *t* + 1, *t* + 2, *t* + 3 and *t* + 4 (i.e., 2008, 2009, 2010, 2011) to determine the rank of insured in these years. However, one might question whether insured in year *t* have (all) information concerning the upcoming years. An alternative approach would be to use only the information from year *t* and *t* − 1 to determine the rank of insured in future years. With the first approach, we would overestimate adverse selection, while with the second approach we would underestimate adverse selection into multiple-year contracts. After all, research shows that substantial consumer information surplus exists also for multiple-year contracts [32], meaning that insured do have some information regarding their future healthcare expenses that may not be picked up by administrative information from year *t* − 1. Therefore, and for reasons of simplicity, we determine the rank of insured for all years using the first mentioned approach for both the ex-ante and ex-post differentiation. In “Appendix 3” we will show that the possible overestimation of adverse selection under this approach has no impact on the main conclusions of this paper.

### The ex-ante differentiation

For the ex-ante differentiation, we simulate a distribution of insured over the different contract periods. We assume that an insurer wants to determine the premiums in year *t* (i.e., 2007 for the ex-ante differentiation) in case he would offer six different insurance policies: a policy without a voluntary deductible and five policies with different contract periods for the voluntary deductible, i.e., 1, 2, 3, 4 or 5 years. Remember that for all years we have ranked insured according to their predicted expenses in that year. We assume that the half of the sample with the lowest predicted expenses in year *t* opts for an insurance policy with a deductible. To then determine who will opt for which multi-year contract, we sum the rank of the insured over the different contract periods. In other words, for a 2-year contract, we sum the rank of insured in year *t* and *t* + 1, and for a 3-year contract, we sum the rank of insured in year *t*, *t* + 1 and *t* + 2, etc. From the half of the sample that is assumed to opt for a policy with a deductible, the quintile with the lowest sum-rank for a 5-year contract is assumed to opt for that policy. From the remaining 40 % of insured opting for a deductible, the quarter with the lowest sum-rank for a 4-year contract is assumed to opt for that policy. From the remaining 30 % of insured opting for a deductible, the third with the lowest sum-rank for a 3-year contract is assumed to opt for that policy. From the remaining 20 % of insured opting for a deductible, the half of insured with the lowest sum-rank for a 2-year contract is assumed to opt for that policy and the other half is assumed to opt for a 1-year contract with a deductible. Note that this process does not include the simulation of a behavioral effect where insured would also opt for a long contract period even if they incidentally expect high healthcare expenses in 1 year during the contract period. In the end, this process provides us with a distribution of insured in year *t* where a group of 50 % of the insured does not opt for a deductible and five groups of 10 % do opt for a deductible with respectively a 1-, 2-, 3-, 4- or 5-year contract period. For this distribution of insured, we subsequently determine the premiums per insurance policy.

### The ex-post differentiation

For the ex-post differentiation, we simulate a distribution of insured in year *t* (i.e., 2011 for the ex-post differentiation) based upon the number of previous consecutive years insured have opted for a voluntary deductible. For this differentiation, we assume that an insurer decreases the premium with each additional consecutive year an insured has opted for a voluntary deductible since year *t* − 4 (i.e., 2007 for this differentiation). Two scenarios are simulated depending on the potential behavioral effect of this differentiation.

In scenario I, we assume that the differentiation of the premium has no effect on the decision to opt for a voluntary deductible, which is contrary to what would be expected (and was expected by the Dutch government). Insured only opt for a deductible if they belong to the half of the sample with the lowest predicted expenses. Looking back from year *t*, we determine for insured who are assumed to opt for a deductible in year *t* the number of previous consecutive years they are assumed to opt for a voluntary deductible as well.

In scenario II, we assume a moderate behavioral effect of the differentiation of the premium. We assume that insured are willing to keep the voluntary deductible for 1 year they expect (high) healthcare expenses (i.e., belong to the half of the sample with the highest predicted healthcare expenses). This means for instance that if an insured is assumed to opt for a voluntary deductible in year *t* − 4, he will also opt for a deductible in year *t* − 3, irrespective of his rank in that year. The insured is thereafter assumed to opt out of the voluntary deductible in year *t* − 2 only if he belongs to the half of the sample with the highest predicted healthcare expenses in both year *t* − 3 and *t* − 2, etc. In the end, this process provides us with a scenario where some retention of the voluntary deductible results from the differentiation of the premium, but where insured also opt out of the voluntary deductible if they for instance incur a chronic disease. The simulation process for these scenarios results in a distribution of insured in year *t* over six groups: insured without a voluntary deductible, insured with a voluntary deductible with different numbers of previous consecutive years they have opted for deductible (i.e., 0, 1, 2, 3, or 4 previous consecutive years).

### Composition of the premium

After the distribution of insured over the deductible options for both the ex-ante and ex-post differentiation in year *t* are simulated, we calculate the premium for each of the six aforementioned groups per distribution. For the analyses, we assume a voluntary deductible of €1000. The average healthcare expenses per individual ($\mathop {\overline{\text{HCE}} }\limits^{{}}$) in the dataset are €1894^9^ in all years. The premium is determined using Eqs. (1a) and (1b) for respectively insured without and with a voluntary deductible:

1a $$P_{\text{NVD}} = \mathop {\overline{\text{IC}}_{\text{NVD}} } \limits^{{}} + \mathop {\overline{\text{REP}}_{\text{NVD}} }\limits^{{}}$$

1b $$P_{\text{VD}} = \mathop {\overline{\text{IC}}_{\text{VD}} }\limits^{{}} + \mathop {\overline{\text{REP}}_{\text{VD}} }\limits^{{}}$$

where *P* is the premium, NVD indicates insured without a voluntary deductible, VD indicates insured with a voluntary deductible (with either different contract periods or different numbers of previous years they have opted for a deductible), $\mathop {\overline{\text{IC}} }\limits^{{}}$ are the average insurance claims and $\mathop {\overline{\text{REP}} }\limits^{{}}$ represents the average risk equalization payment. Without any risk equalization, the equations show that the premium equals the average insurance claims in the group. With risk equalization, however, the premium is affected by the risk equalization payment, which is determined for insured without a voluntary deductible using Eq. (2a)^10^:

2a $$\mathop {\overline{\text{REP}}_{\text{NVD}} }\limits^{{}} = \frac{x}{100}\left[ {\mathop {\overline{\text{HCE}} }\limits^{{}} - \mathop {\overline{\text{IC}}_{\text{NVD}} }\limits^{{}} } \right]$$

where *x* indicates the quality of the risk equalization model^11^ and $\mathop {\overline{\text{HCE}} }\limits^{{}}$ indicates the average healthcare expenses in the data without any cost-sharing arrangements. Due to the voluntary deductible, the risk equalization payment for insured with a voluntary deductible is different to that of insured without a voluntary deductible and determined using Eq. (2b):

2b $$\mathop {\overline{\text{REP}}_{\text{VD}} }\limits^{{}} = \frac{x}{100}\left[ {\mathop {\overline{\text{HCE}} }\limits^{{}} - \left( {\mathop {\overline{\text{IC}}_{\text{VD}} }\limits^{{}} { + }\mathop {\overline{\text{MHR}}_{\text{VD}} }\limits^{{}} { + }\mathop {\overline{\text{OOP}}_{\text{VD}} }\limits^{{}} } \right)} \right]$$

where $\mathop {\overline{\text{MHR}} }\limits^{{}}$ and $\mathop {\overline{\text{OOP}} }\limits^{{}}$ respectively indicate the average moral hazard reduction and the average out-of-pocket expenses for the group of insured with a voluntary deductible (for different contract periods or in multiple consecutive years) resulting from the deductible. After the risk equalization payment for the different groups of insured is determined, the premiums can be calculated using Eqs. (1a) or (1b) depending on whether the insured has a deductible or not.

In order to determine the differentiated premiums that can be offered by insurers, we need to know (1) the average healthcare expenses in the data, (2) the average insurance claims, the average moral hazard reduction and the average out-of-pocket expenses for the different groups of insured, and (3) the quality of the risk equalization model. Firstly, the average healthcare expenses in the data are already mentioned and equal €1894. Secondly, for the average insurance claims for insured who are not assumed to opt for a deductible, we use the healthcare expenses in the data. Since no cost-sharing arrangements are in place in our data, the healthcare expenses in the data for insured who are assumed to opt for a deductible include a moral hazard reduction and out-of-pocket expenses they would have in case of a voluntary deductible. Many researchers have studied the reduction of healthcare expenses resulting from voluntary deductibles [e.g., 2, 3, 13, 18, 30]. For our simulations, we use the reduction as determined in the study by Trottmann et al. [30] since the researchers of this recent study corrected for the selection effect that arises when taking out voluntary deductibles. Consequently, the healthcare expenses in the data of insured with a voluntary deductible are reduced by 22.6 % due to the voluntary deductible. The size of the out-of-pocket expenses is determined as the healthcare expenses after the moral hazard reduction in the interval [0:1000]. The insurance claims for insured with a voluntary deductible are then determined as the healthcare expenses in the data minus the moral hazard reduction and minus the out-of-pocket expenses. For instance, an insured with healthcare expenses of “€1250 in the data” and a voluntary deductible of €1000 will have a moral hazard reduction of €283 (€1250 × 0.226), out-of-pocket expenses of €967 (€1250 − €283) and no insurance claims (€1250 − €283 − €967). However, if an insured who opts for a voluntary deductible of €1000 has healthcare expenses of “€2500 in the data”, the moral hazard reduction will be €566 (€2500 × 0.226), the out-of-pocket expenses will be €1000 (€2500 − €566 = €1934) and the insurance claims equal €934 (€2500 − €566 − €1000). Thirdly, to determine the effect of risk equalization on the premium, Van Kleef et al. [35] show that equalization based upon region, age and gender, and equalization based upon demographic factors, PCGs and DCGs respectively reduce the adverse selection component of the premium rebate for the highest Swiss voluntary deductibles with respectively 47 and 74 % in 2006. For our simulations, we therefore study the effect of no risk equalization, perfect risk equalization and the two models used in the research by Van Kleef et al. [35]. Note that due to extensive research, risk equalization schemes have become more sophisticated and that the Dutch scheme of 2015 is already more sophisticated than the 74 % model studied by Van Kleef et al. [35].

## Results

### Situation without differentiation

Since different percentages of insured who are assumed to opt for a voluntary deductible can be studied, Table 1 shows the results under the assumption that 5, 25, or 50 % of the sample with the lowest predicted expenses would opt for a voluntary deductible of €1000 without any differentiation of the premium without any risk equalization. Three insights are drawn from this table. Firstly, the table shows that both the premiums and premium rebates increase with the percentage of insured opting for a deductible. The latter is partially due to the increase of the moral hazard reduction and out-of-pocket expenses, but largely due to the increase in adverse selection. Secondly, in all three cases, the premium rebate is larger than the voluntary deductible itself.^13^ Thirdly, the premium rebate in general exists for the largest part of an adverse selection component (i.e., 88 % of the premium rebate if 50 % of the insured would opt for a voluntary deductible).

**Table 1:** Composition of the premium (*P*) in 2007 for an insurance policy with a voluntary deductible of €1000 for different percentages of insured with the lowest predicted healthcare expenses who are assumed to opt for a voluntary deductible, without any differentiation of the premium, without any risk equalization

%		$\mathop {\overline{\text{HCE}} }\limits^{{}}$	$\mathop {\overline{\text{IC}} }\limits^{{}}$ ^a^	$\mathop {\overline{\text{MHR}} }\limits^{{}}$	$\mathop {\overline{\text{OOP}} }\limits^{{}}$	P^a^
5 % opts for a voluntary deductible						
95	NVD	€1894	€1981			€1981
5	VD		€46	€51	€129	€46
25 % opts for a voluntary deductible						
75	NVD	€1894	€2417			€2417
25	VD		€75	€74	€176	€75
50 % opts for a voluntary deductible						
50	NVD	€1894	€3289			€3289
50	VD		€128	€113	€257	€128

$\mathop {\overline{\text{HCE}} }\limits^{{}}$, average healthcare expenses; $\mathop {\overline{\text{IC}} }\limits^{{}}$, average insurance claims; $\mathop {\overline{\text{MHR}} }\limits^{{}}$, average moral hazard reduction; $\mathop {\overline{\text{OOP}} }\limits^{{}}$, average out-of-pocket expenses; P, premium; NVD, insured without a voluntary deductible; VD, insured with a voluntary deductible

^a^Note that since a situation without risk equalization is shown, no risk equalization payment is in place and the insurance claims equal the premium

### The ex-ante differentiation

Remember that, for the ex-ante differentiation, we want to determine the premiums in year *t* (i.e., 2007 for this differentiation) in case an insurer would offer six insurance policies: a policy without a voluntary deductible and five policies with different contract periods for the voluntary deductible. Table 2 shows the results of this simulation without any risk equalization and provides three insights. Firstly, the table shows that the premium could decrease with the duration of the contract. Secondly, the premium for insured with a 1-year contract for the deductible is much higher compared to the situation without differentiation. Thirdly, the table shows that the moral hazard reduction for a 1-year contract and the out-of-pocket expenses for both a 1- and 2-year contract are larger compared to the situation without differentiation.

**Table 2:** Ex-ante option. Composition of the premium for an insurance policy with a differentiation of the premium to the duration of the contract period for a voluntary deductible of €1000 in year *t* without any risk equalization

%		$\mathop {\overline{\text{HCE}} }\limits^{{}}$	$\mathop {\overline{\text{IC}} }\limits^{{}}$ ^a^	$\mathop {\overline{\text{MHR}} }\limits^{{}}$	$\mathop {\overline{\text{OOP}} }\limits^{{}}$	P^a^
50	NVD	€1894	€3289			€3289
50	VD		€128	€113	€257	€128
10	VD 1-year contract		€573	€360	€656	€573
10	VD 2-year contract		€35	€87	€262	€35
10	VD 3-year contract		€19	€56	€173	€19
10	VD 4-year contract		€12	€39	€123	€12
10	VD 5-year contract		€1	€22	€72	€1

$\mathop {\overline{\text{HCE}} }\limits^{{}}$, average healthcare expenses; $\mathop {\overline{\text{IC}} }\limits^{{}}$, average insurance claims; $\mathop {\overline{\text{MHR}} }\limits^{{}}$, average moral hazard reduction; $\mathop {\overline{\text{OOP}} }\limits^{{}}$, average out-of-pocket expenses; P, premium; NVD, insured without a voluntary deductible; VD, insured with a voluntary deductible

^a^Note that since a situation without risk equalization is shown, no risk equalization payment is in place and the insurance claims equal the premium

### The ex-post differentiation

Remember that for the ex-post differentiation, scenario I simulated a situation without any behavioral effect. The upper part of Table 3 shows the results for scenario I without any risk equalization and provides three insights. Firstly, the table shows that the premium could decrease with the number of previous consecutive years insured have opted for a deductible. Secondly, it shows that compared to the situation without differentiation, the premium is higher for the first three consecutive years. Thirdly, the table shows that the moral hazard reduction and out-of-pocket expenses are larger for the first four consecutive years opting for a voluntary deductible compared to the situation without differentiation.

**Table 3:** Ex-post option. Composition of the premium for an insurance policy with a differentiation of the premium to the number of previous consecutive years an insured has opted for a voluntary deductible of €1000 in year *t* without any risk equalization

%		$\mathop {\overline{\text{HCE}} }\limits^{{}}$	$\mathop {\overline{\text{IC}} }\limits^{{}}$ ^a^	$\mathop {\overline{\text{MHR}} }\limits^{{}}$	$\mathop {\overline{\text{OOP}} }\limits^{{}}$	P^a^
SCENARIO I: no behavioral effect						
50	NVD	€1894	€3287			€3287
50	VD		€133	€113	€254	€133
9.1	1 VD		€238	€181	€381	€238
6.0	2 VD		€192	€152	€327	€192
4.7	3 VD		€141	€126	€291	€141
3.5	4 VD		€120	€114	€268	€120
26.7	5 VD		€85	€79	€186	€85
SCENARIO II: moderate behavioral effect						
40.9	NVD	€1894	€3584			€3584
59.0	VD		€262	€163	€296	€262
4.6	1 VD		€287	€208	€423	€287
4.6	2 VD		€502	€281	€457	€502
3.5	3 VD		€349	€219	€400	€349
6.4	4 VD		€332	€200	€350	€332
39.9	5 VD		€213	€134	€245	€213

$\mathop {\overline{\text{HCE}} }\limits^{{}}$, average healthcare expenses; $\mathop {\overline{\text{IC}} }\limits^{{}}$, average insurance claims; $\mathop {\overline{\text{MHR}} }\limits^{{}}$, average moral hazard reduction; $\mathop {\overline{\text{OOP}} }\limits^{{}}$, average out-of-pocket expenses; P, premium; NVD, insured without a voluntary deductible; VD, insured with a voluntary deductible (the number corresponds to the number of consecutive years)

^a^Note that since a situation without risk equalization is shown, no risk equalization payment is in place and the insurance claims equal the premium

In scenario II, insured are assumed to keep the voluntary deductible during 1 year they expect (high) healthcare expenses incentivized by the premium differentiation. The bottom part of Table 3 shows the results for scenario II without any risk equalization and provides three insights. Firstly, the results show that the premium would increase sharply for the second consecutive year compared to the first year opting for a deductible and that the premium would thereafter considerably decrease. This would imply that only after the second consecutive year, offering a decreasing premium with the number of years insured have opted for a deductible would be feasible in case of no risk equalization. The increase in premium for the second consecutive year follows from the retention of the voluntary deductible in that year even if (high) healthcare expenses are expected, meaning that insured who expect (high) healthcare expenses for the second consecutive year opting for a deductible are included in that group. Secondly, compared to a situation without differentiation, the premium is only lower for the fifth consecutive year opting for a voluntary deductible. Thirdly, the moral hazard reduction and out-of-pocket expenses are larger for the first four consecutive years opting for a deductible compared to the situation without differentiation of the premium. Compared to scenario I, the moral hazard reduction and out-of-pocket expenses are larger for all groups, except for the out-of-pocket expenses for insured with a voluntary deductible for five consecutive years.

### Risk equalization

**Fig. 2:**
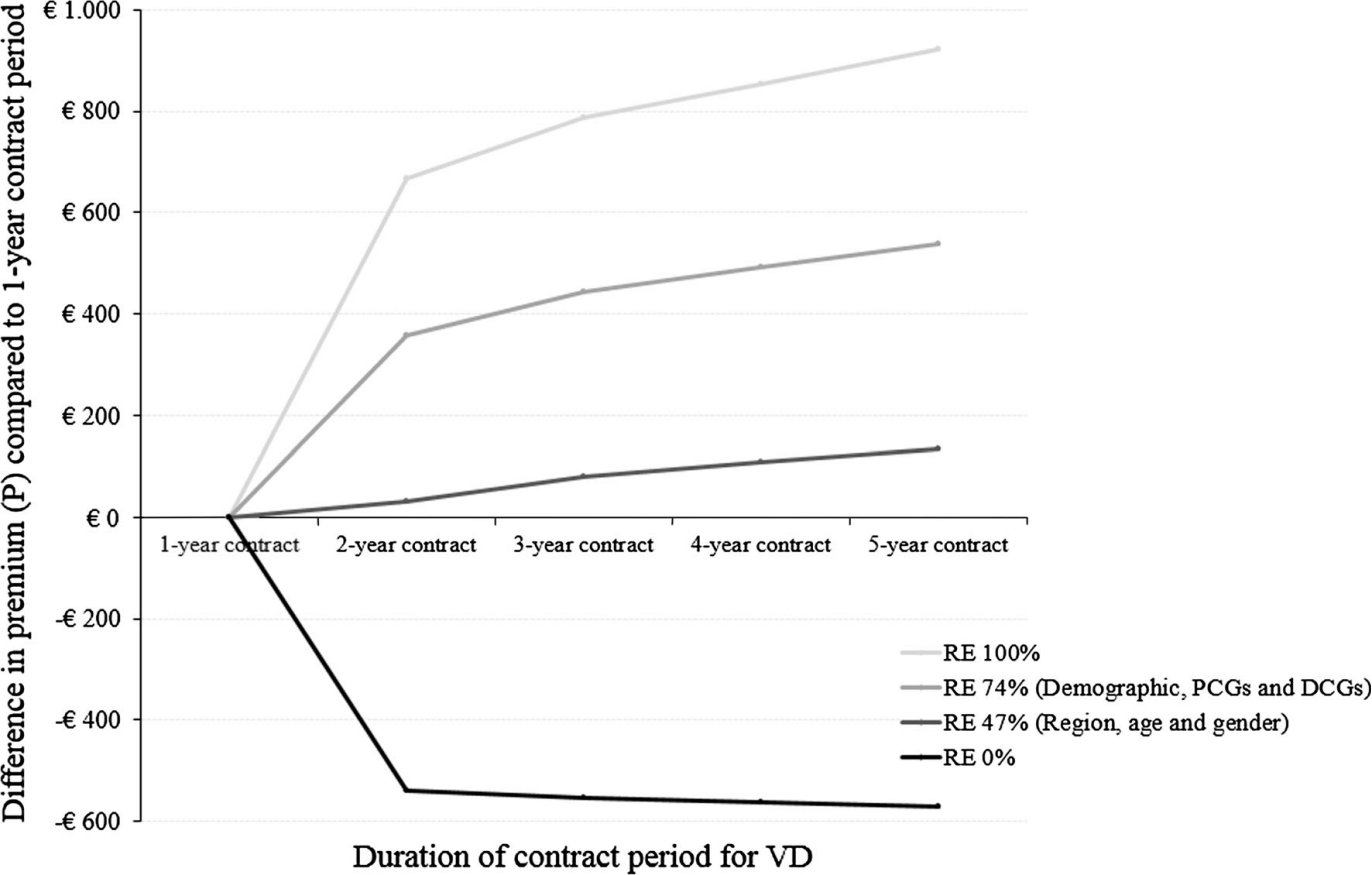
Difference in premium (*P*) compared to a 1-year contract for the voluntary deductible (VD) for risk equalization models that equalize either 0, 47, 74, or 100 % of the difference in predicted expenses between low-risk and high-risk individuals where the distribution of insured in year *t* (i.e., 2007) is determined according to the ex-ante differentiation of the premium

Table 4 provides the estimated ex-ante differentiated premiums in case of perfect risk equalization. It shows that, as a result of perfect risk equalization, the premium is expected to increase with the duration of the contract. Furthermore, Table 4 shows that only insured with a 1-year contract period for the deductible pay a lower premium compared to the situation without differentiation of the premium. Figure 2 additionally shows the premium patterns for an ex-ante differentiation compared to a 1-year contract for the voluntary deductible for risk equalization models that equalize 0 %, 47 % (i.e., based upon region, age, and gender), 74 % (i.e., based upon demographic factors, PCGs and DCGs) or 100 % of the differences in predicted expenses between low-risk and high-risk individuals. It shows that a decreasing premium with the duration of the contract for which the deductible holds is only feasible without any risk equalization.

**Table 4:** Ex-ante option. Composition of the premium for an insurance policy with a differentiation of the premium to the duration of the contract period for a voluntary deductible of €1000 in year *t* with perfect risk equalization

%		$\mathop {\overline{\text{IC}} }\limits^{{}}$	$\mathop {\overline{\text{REP}} }\limits^{{}}$	P
50	NVD	€3289	€−1395	€1894
50	VD	€128	€1396	€1524
10	VD 1 year contract	€573	€305	€878
10	VD 2 year contract	€35	€1510	€1545
10	VD 3 year contract	€19	€1646	€1665
10	VD 4 year contract	€12	€1720	€1732
10	VD 5 year contract	€1	€1799	€1800

$\mathop {\overline{\text{IC}} }\limits^{{}}$, average insurance claims; $\mathop {\overline{\text{REP}} }\limits^{{}}$, risk equalization payment, determined according to Eq. (2a) or (2b) given the $\mathop {\overline{\text{MHR}} }\limits^{{}}$ and $\mathop {\overline{\text{OOP}} }\limits^{{}}$ shown in Table 2; P, premium; NVD, insured without a voluntary deductible; VD, insured with a voluntary deductible

**Fig. 3:**
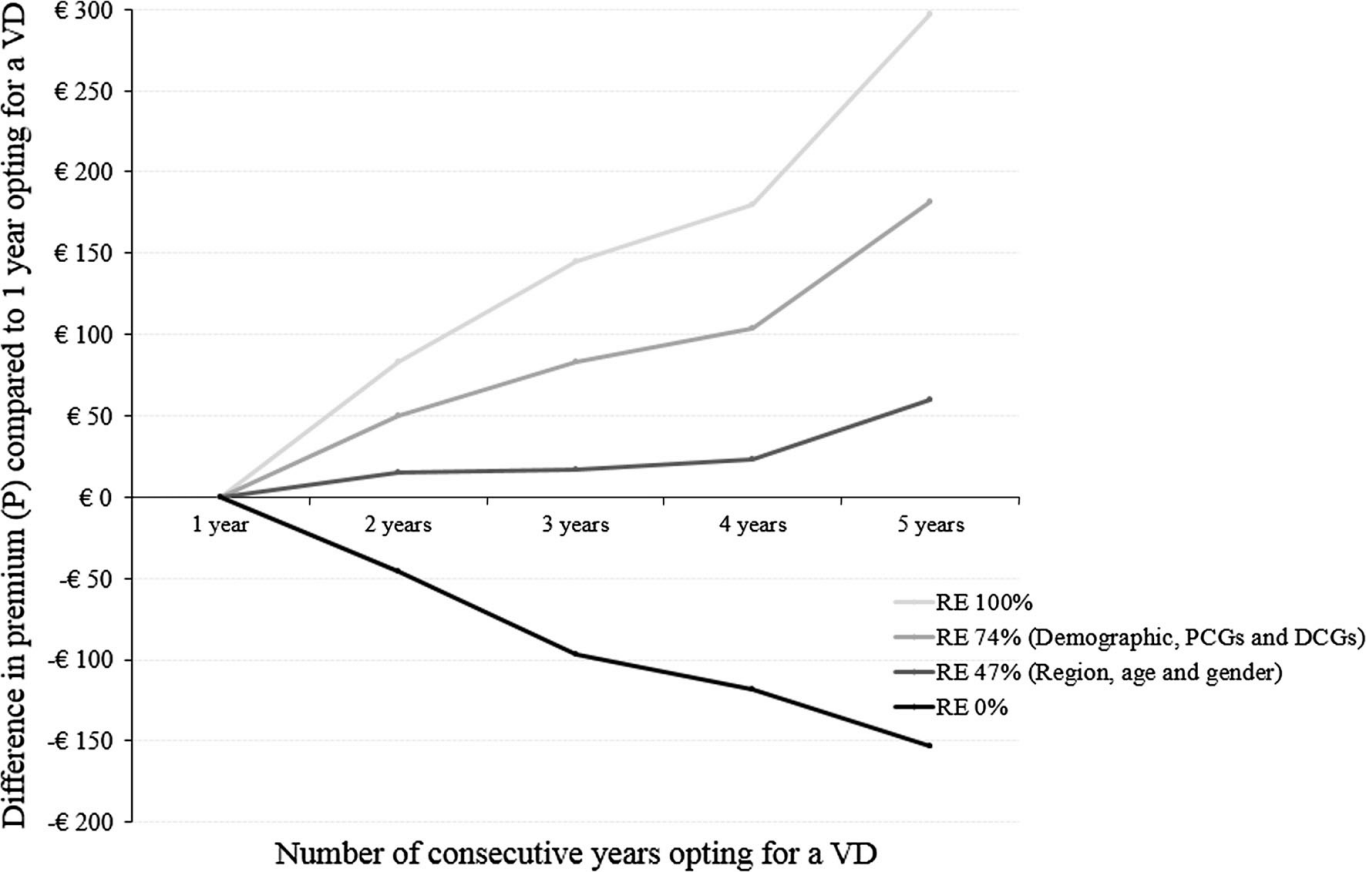
Difference in premium (*P*) compared to 1 year opting for a voluntary deductible (VD) for risk equalization models that equalize either 0, 47, 74, or 100 % of the difference in predicted expenses between low-risk and high-risk individuals where the distribution of insured in year *t* (i.e., 2011) is determined according to the ex-post differentiation of the premium for scenario I (no behavioral effect)

**Fig. 4:**
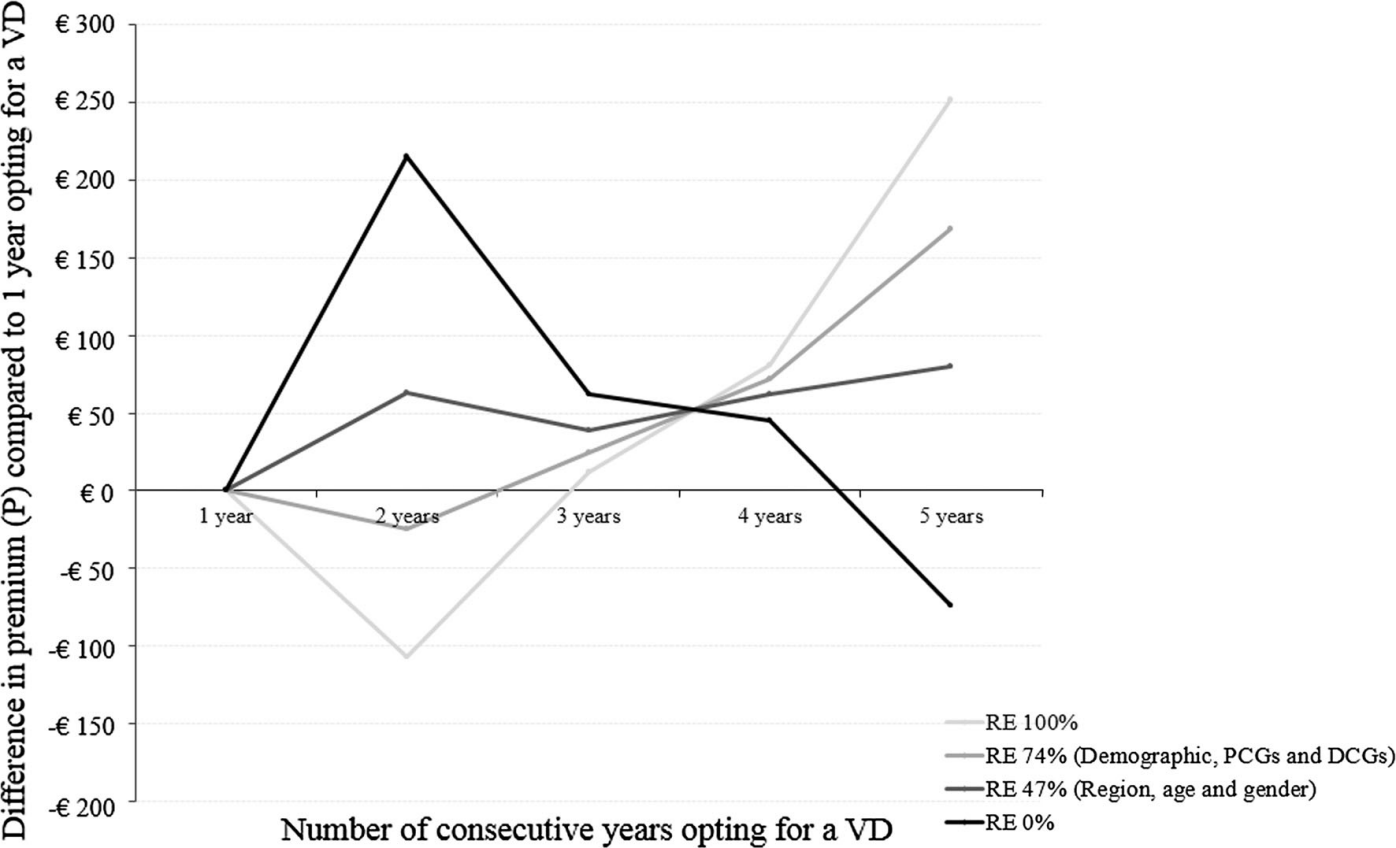
Difference in premium (*P*) compared to 1 year opting for a voluntary deductible (VD) for risk equalization models that equalize either 0, 47, 74, or 100 % of the difference in predicted expenses between low-risk and high-risk individuals where the distribution of insured in year *t* (i.e., 2011) is determined according to the ex-post differentiation of the premium for scenario II (moderate behavioral effect)

Table 5 provides the estimated ex-post differentiated premiums in case of perfect risk equalization. For scenario I (i.e., without any behavioral effect), the table shows that the premium is expected to increase with the number of years insured have opted for a deductible. For scenario II (i.e., with a moderate behavioral effect), the table shows that the premium could decrease between the first and second consecutive year insured have opted for a deductible, but that it would thereafter increase with the number of consecutive years insured have opted for a voluntary deductible. In both scenarios, only insured who opt for a voluntary deductible for the fifth consecutive year are offered a higher premium compared to the situation without differentiation of the premium. Figures 3 and 4 additionally show the premium patterns for an ex-post differentiation for, respectively, scenario I and II compared to 1 year opting for a voluntary deductible for risk equalization models that equalize 0, 47, 74, or 100 % of the differences in predicted expenses between low-risk and high-risk individuals. Figure 3 for scenario I confirms that only without any risk equalization the premium could decrease with the number of consecutive years insured have opted for a deductible. Figure 4 for scenario II shows that the premium could only decrease for the second consecutive year insured have opted for a voluntary deductible in case of perfect or sophisticated (i.e., 74 %) risk equalization. But thereafter, the premium would substantially increase with the number of consecutive years insured have opted for a deductible. Furthermore, the premium could only decrease from the second consecutive year insured have opted for a deductible in a situation without risk equalization. It might therefore not be that surprising that none of the Dutch insurers utilizes the option to ex-post differentiated the premium. Overall, under the assumptions made within this paper, these results imply that, due to risk equalization, it does not seem likely that insurers in Germany, Switzerland, the Netherlands, or the US could offer a decreasing premium if it is either differentiated to the duration of the contract for which the voluntary deductible holds or the number of previous consecutive years insured have opted for a voluntary deductible.

**Table 5:** Ex-post option. Composition of the premium for an insurance policy with a differentiation of the premium to the number of previous consecutive years an insured has opted for a voluntary deductible of €1000 in year *t* with perfect risk equalization

%		$\mathop {\overline{\text{IC}} }\limits^{{}}$	$\mathop {\overline{\text{REP}} }\limits^{{}}$	P
SCENARIO I: no behavioral effect				
50	NVD	€3287	€−1393	€1894
50	VD	€133	€1394	€1507
9.1	1 VD	€238	€1094	€1299
6	2 VD	€192	€1223	€1389
4.7	3 VD	€141	€1336	€1456
3.5	4 VD	€120	€1392	€1495
26.7	5 VD	€85	€1544	€1617
SCENARIO II: moderate behavioral effect				
40.9	NVD	€3584	€−1690	€1894
59	VD	€262	€1173	€1435
4.6	1 VD	€287	€976	€1263
4.6	2 VD	€502	€654	€1156
3.5	3 VD	€349	€926	€1275
6.4	4 VD	€332	€1012	€1344
39.9	5 VD	€213	€1302	€1515

$\mathop {\overline{\text{IC}} }\limits^{{}}$, average insurance claims; $\mathop {\overline{\text{REP}} }\limits^{{}}$, risk equalization payment, determined according to Eq. (2a) or (2b) given the $\mathop {\overline{\text{MHR}} }\limits^{{}}$ and $\mathop {\overline{\text{OOP}} }\limits^{{}}$ shown in Table 3; P, premium; NVD, insured without a voluntary deductible; VD, insured with a voluntary deductible (the number corresponds to the number of consecutive years)

## Conclusions

Voluntary deductibles are implemented in regulated insurance markets such as Germany, Switzerland, the Netherlands, and the US to counteract moral hazard. However, the moral hazard reduction resulting from these deductibles could be mitigated by adverse selection, since insured only opt for a deductible if their expected out-of-pocket expenses under the deductible are smaller than the premium rebate. Insurers in regulated markets may reduce this adverse selection by differentiating the premium according to either the duration of the contract for which the voluntary deductible holds (ex-ante approach) or the number of previous years insured have opted for a voluntary deductible (ex-post approach). It can be hypothesized that, the longer the period for which the voluntary deductible holds or the more previous years an insured has opted for a voluntary deductible, the lower the premium will be. This would incentivize insured to opt for a voluntary deductible for a longer period or for another consecutive year. To determine the premiums that could be offered in case of such differentiations, we simulated the distribution of insured over the deductible options for both the ex-ante and ex-post differentiation. Thereafter, we calculated the premiums based upon the insurance claims and a risk equalization payment. The results show that only without risk equalization insurers would be able to offer a decreasing premium with the duration of the contract or with the number of previous consecutive years insured have opted for a voluntary deductible. With moderate, sophisticated, or perfect risk equalization, the premiums are expected to increase for both the ex-ante and ex-post differentiation. These results are due to the fact that as either the duration of the contract or the number of years insured have opted for a voluntary deductible increases, the insurance claims decrease but the risk equalization payments increase, which overall increases the premium. In sum, under the assumptions made in this paper, the results imply that, due to risk equalization, it seems unlikely that insurers in Germany, Switzerland, the Netherlands, or the US can offer premiums that decrease with either the duration of the contract for which the voluntary deductible holds or the number of previous consecutive years insured have opted for a voluntary deductible. Given these findings, we are sceptical about the feasibility of these strategies to counteract adverse selection.

## Discussion

### General discussion points

This section provides four general discussion points regarding the representativeness of our data, the sample sizes of the groups under study, the way risk equalization is taken into account and the omission of the loading fee in our analyses. Firstly, as mentioned when discussing the data, the average health in the sample is somewhat worse compared to the Dutch population. This implies that the absolute premiums presented in this paper are probably higher than they would be in the Dutch population. The effect of this limitation on relative premiums (i.e., the increase or decrease of the premium with the duration of the contract period or the number of previous consecutive years opting for a voluntary deductible) and our conclusions, however, will be minor since deductible choice is simulated relative to spending and characteristics in our sample and not relative to an absolute benchmark. Furthermore, it seems most likely that if insurers would want to differentiate the premiums for their health insurance policies, they would use their own data to determine these premiums. Nevertheless, we emphasize that our absolute results are not generalizable to the entire (Dutch) population. In order to achieve more generalizable results, a more representative dataset would be necessary.

Secondly, some of the groups resulting from our simulations are quite small (e.g., 3.5 % of the sample) and the results of these groups could be affected by a few insured in the data with very large insurance claims. However, only 176 insured (i.e., 0.02 %) and 17 insured (i.e., 0.002 %) in the data had healthcare expenses, respectively, larger than €100,000 or €200,000 in 2011. Sensitivity checks where these insured are omitted show that the absolute premiums change only marginally and that the relative premiums remain unchanged.

Thirdly, contrary to our analyses, cost reductions resulting from the voluntary deductible (i.e., moral hazard reduction and out-of-pocket expenses) are in Switzerland, Germany, and the Netherlands partially captured by risk equalization and can consequently not be fully reflected into the premium rebate [36]. This implies that the premium for insured with a deductible would be higher compared to our paper, but it does not affect the relative premiums found in this paper.

Fourthly, this paper only studied insurance claims and disregarded the insurer’s loading fee. Although the loading fee does not constitute a large part of the premium, it could affect the premium, the premium rebate and risk equalization if the average loading fee differs between insured with and without a voluntary deductible. For instance, administration costs differ between these groups since insured with a deductible do not send their bills to the insurer before the total amount exceeds the deductible. Consequently, the insurer does not have to handle the bills of these insured [34]. Even in a system where most bills are settled between the insurer and the provider, such as in the Netherlands, a difference in administration costs between insured who do and do not opt for a deductible can exist. One can hypothesize that, since upcoding is a serious problem [29], insurers spend a lot of time verifying the received bills. Since insured with a voluntary deductible on average use less healthcare services and therefore file less bills compared to insured without a deductible, it could be assumed that verifying the bills of the latter group is more expensive. In a market without any (or with poor) risk equalization, this could imply that the premiums could potentially decrease for insured with a voluntary deductible compared to the simulations showed within this paper due to smaller administrative costs. However, further research regarding the effect of the loading fee on the premium—also in case of an ex-ante or ex-post differentiation—is necessary.

### Empirical assumptions

This section provides five discussion points regarding the assumptions in our empirical simulations. Firstly, we based the assumption of which insured opt for a voluntary deductible solely on the predicted healthcare expenses. However, research shows that other determinants than the predicted expenses affect the decision to opt for a deductible as well, such as loss aversion, risk attitude, ambiguity aversion, debt aversion and omission bias [38]. As a result of these factors, it could be expected that fewer insured (i.e., less than 50 %) opt for a voluntary deductible. As shown in Table 1, fewer insured opting for a deductible affects the absolute premium, but sensitivity analyses showed that the relative premiums for both the ex-ante and ex-post differentiation would not be affected.

Secondly, since we simulate who opts for a deductible based upon predicted expenses, we are unable to incorporate planned medical decisions not identified by the explanatory variables used in our estimation models, such as pregnancy. In our simulations, insured with unidentified planned medical decisions might opt for a deductible, while in practice they would not due to (high) expected healthcare expenses. We therefore may underestimate adverse selection into the different deductible options.

Thirdly, we do not study expenses for different types of healthcare services, but only use total healthcare expenses. Gerfin et al. [13] show that deductibles affect different types of healthcare differently, where the decrease in healthcare expenses due to the deductible is most pronounced for inpatient care and prescription drugs. This could impact the insured’s decision to opt for a voluntary deductible, which is not taken into account in our analyses.

Fourthly, we assume very strong adverse selection into the different deductible options: the entire half of the sample with the lowest predicted expenses opts for a voluntary deductible. A sensitivity check for scenario I of the ex-post differentiation shows that if this assumption is relaxed (i.e., one in two insured belonging to the half of the sample with the lowest predicted expenses randomly opts for a deductible in year *t*), the premiums still increase with the number of consecutive years insured have opted for a voluntary deductible in case of perfect risk equalization.

Fifthly, for the ex-ante differentiation, we determine the rank of insured in the upcoming years using information from these years. It might, however, be questionable how much information insured actually have on future expenses. Therefore, we performed a sensitivity check for the ex-ante differentiation using only the information known at the start of year *t* to rank insured (see the results in “Appendix 3”). This approach implies an underestimation of adverse selection, since research shows that insured do have some information on future healthcare expenses [32]. The sensitivity check shows that, although the absolute premiums are somewhat different, the relative premiums show the same pattern as with the approach used within this paper: without risk equalization, the premiums decrease with the duration of the contract period and with risk equalization, the premiums increase with the duration of the contract period.

### Market dynamics

Our results show the first-order effects (i.e., the premiums when starting to offer insurance policies with ex-ante or ex-post differentiated premiums) and disregard any market dynamics. This section elaborates on these market dynamics for markets without risk equalization since the results already showed that ex-ante or ex-post differentiated decreasing premiums are impossible with sophisticated or perfect risk equalization.

Regarding the ex-ante differentiation, if insurer ‘A’, for instance, would offer these six health insurance policies, insured would have a large incentive to opt for a policy with a contract period longer than 1 year due to the decreasing premium. However, if a competitor would not offer differentiated premiums, his premium for a policy with a voluntary deductible would be much lower (i.e., €128 compared to €573; see Table 2). If insurer ‘A’ would be able, due to optimal marketing, to attract the healthiest insured away from his competitor into the long contract policies, it might indeed be attractive to offer ex-ante differentiated premiums. The reason is that in that case, his competitor has set the premium for a policy with a voluntary deductible too low and must increase it. Additionally, since the results only show the first-order effects, the estimated premiums could change over time when the contract expires and insured once again get to choose between the different deductible options. As with the ex-post approach, it could be that insured are willing to accept (high) healthcare expenses during 1 year of their contract period. Further research into this dynamic behavioral effect for insurance markets without risk equalization would be necessary to provide insights into the resulting premium patterns.

Regarding the ex-post differentiation, scenario I (Table 3) especially shows the first-order effects. In that case, insurer ‘B’, for instance, could offer these six health insurance policies, but he could also decide to combine two or three insurance policies. Note that, as with the ex-ante differentiation, a competitor of insurer ‘B’ who might not offer differentiated premiums, might be less expensive than insurer ‘B’. Again, in that case, insurer ‘B’ should try to attract the healthiest insured away from his competitors into the policies for insured who opted for a deductible in more consecutive years. From scenario I, scenario II could be interpreted as the situation after 5 years (i.e., year *t* + 5) with some market dynamics simulated as a moderate behavioral effect of the decreasing premium on the insured’s decision to opt for a deductible. It already shows that decreasing premiums could only be offered upward of two consecutive years, but further research into market dynamics and behavioral effects in insurance markets without risk equalization would be necessary to provide further insight into the resulting premium patterns.

## Appendix 1: Several models to estimate the predicted healthcare expenses and accordingly rank insured

Several models are tested to determine the predicted healthcare expenses of individuals in year *t* based upon their background characteristics.

1. Ordinary least squares
2. Ordinary least squares with a log transformation of healthcare expenses
3. Generalized linear model with a gamma distribution and a log link
4. Generalized linear model with a gamma distribution and a power −1 link
5. Generalized linear model with a gamma distribution and a power 0.5 link
6. Generalized linear model with a normal distribution and a log link.

Table 6 shows the Spearman correlation coefficients of the predicted healthcare expenses with the actual healthcare expenses in year *t* for all models. Table 6 shows these correlation coefficients for both the entire dataset and a subset of the sample. This subset regards the half of insured with the lowest actual healthcare expenses in year *t*. We also determined the correlation coefficient for this subset to see whether our preferred model would also perform well for the healthiest insured since these would be the insured we would select to opt for a voluntary deductible. Since the second model, the OLS with a log transformation of healthcare expenses, has the highest correlation coefficient on both the entire dataset and the subset of the sample, this model is used to determine the predicted healthcare expenses of all insured in year *t* and rank insured accordingly.

**Table 6:** Spearman correlation coefficient of the predicted healthcare expenses according to the different models with the actual healthcare expenses in year *t* for the entire dataset and for the half of insured with the lowest actual healthcare expenses

		Entire dataset	Subset of sample
0	Actual healthcare expenses in year *t*	1.0000	1.0000
1	OLS	0.6857	0.3814
2	OLS with log transformation of THCE	0.7384	0.5192
3	GLM with gamma distribution and log link	0.7327	0.5021
4	GLM with gamma distribution and power −1 link	0.5928	0.4730
5	GLM with gamma distribution and power 0.5 link	0.7326	0.5010
6	GLM with normal distribution and log link	0.6947	0.4356

THCE, total healthcare expenses under basic insurance

## Appendix 2: Independent variables included in statistical model

Table 7 provides a description of the independent variables that are included in the model. The six models mentioned in “Appendix 1” were also tested with the same independent variables as mentioned in Table 7 but with the past total healthcare expenses in year *t* − 1 included as a continuous variable (instead of included as dummy variables for the vigintiles). However, with this operationalization of the past total healthcare expenses, the correlation coefficients were much lower (e.g., the correlation coefficient for the second model was 0.6480).

**Table 7:** Description of the independent variables of the model to determine the predicted healthcare expenses in year *t*

Independent variables	Description	Number of variables in the model
Age/gender	28 Classes (i.e., 14 classes for males and 14 classes for females) with age in 5-year classes starting from 18–24 years up to an age of 84. Insured older than 84 years are also included in a separate risk class.	27^a^
PCG	1 Class to indicate whether an insured is classified into a PCG in year *t*. Individuals are assigned to a PCG when they used at least 180 daily dosages of a specific drug in year *t* − 1.	1
DCG	1 Class to indicate whether an insured is classified into a DCG in year *t*. Individuals are assigned to a DCG when they had a hospital admission in year *t* − 1 for a specific diagnosis.	1
Urbanization	5 Classes to indicate the degree of urbanization in the insured's residential area based upon a four digit zip code	4^a^
Ethnicity	1 Class to indicate whether the insured is native or non-native. Non-native insured include insured with a Turkish, Moroccan and Surinamese descent.	1
Past healthcare expenses	20 Classes that indicate the vigintile of total healthcare expenses under basic insurance the insured incurred in year *t* − 1.	19^a^

^a^The number of variables included in the model is for some independent variables one less than the number of defined classes, because one variable for each independent variable is a reference group for all included dummy variables per independent variable

## Appendix 3: Sensitivity check on the rank of insured for the ex-ante differentiation

See Tables 8 and 9.

**Table 8:** Composition of the premium according to the ex-ante differentiation for a voluntary deductible of €1000 where the rank of insured is determined using only the information known at the beginning of year *t* without any risk equalization

%		$\mathop {\overline{\text{HCE}} }\limits^{{}}$	$\mathop {\overline{\text{IC}} }\limits^{{}}$ ^a^	$\mathop {\overline{\text{MHR}} }\limits^{{}}$	$\mathop {\overline{\text{OOP}} }\limits^{{}}$	P^a^
50	NVD	€1894	€3289			€3289
50	VD		€128	€113	€257	€128
10	VD 1-year contract		€232	€187	€406	€232
10	VD 2-year contract		€162	€139	€314	€162
10	VD 3-year contract		€114	€103	€238	€114
10	VD 4-year contract		€79	€77	€185	€79
10	VD 5-year contract		€53	€57	€143	€53

$\mathop {\overline{\text{HCE}} }\limits^{{}}$, average healthcare expenses; $\mathop {\overline{\text{IC}} }\limits^{{}}$, average insurance claims; $\mathop {\overline{\text{MHR}} }\limits^{{}}$, average moral hazard reduction; $\mathop {\overline{\text{OOP}} }\limits^{{}}$, average out-of-pocket expenses; P, premium; NVD, insured without a voluntary deductible; VD, insured with a voluntary deductible

^a^Note that since a situation without risk equalization is shown, no risk equalization payment is in place and the insurance claims equal the premium

**Table 9:** Composition of the premium according to the ex-ante differentiation for a voluntary deductible of €1000 where the rank of insured is determined using only the information known at the beginning of year *t* with perfect risk equalization

%		$\mathop {\overline{\text{IC}} }\limits^{{}}$	$\mathop {\overline{\text{REP}} }\limits^{{}}$	P
50	NVD	€3289	€−1395	€1894
50	VD	€128	€1396	€1524
10	VD 1 year contract	€573	€1069	€1301
10	VD 2 year contract	€35	€1279	€1441
10	VD 3 year contract	€19	€1439	€1553
10	VD 4 year contract	€12	€1553	€1632
10	VD 5 year contract	€1	€1641	€1694

$\mathop {\overline{\text{IC}} }\limits^{{}}$, average insurance claims; $\mathop {\overline{\text{REP}} }\limits^{{}}$, risk equalization payment, determined according to Eq. (2a) or (2b) given the $\mathop {\overline{\text{MHR}} }\limits^{{}}$ and $\mathop {\overline{\text{OOP}} }\limits^{{}}$ shown in table A3; P, premium; NVD, insured without a voluntary deductible; VD, insured with a voluntary deductible

## Footnotes

1 Zweifel [42] has studied the effect of bonuses (instead of deductibles used as a sanction method) in West Germany comparing three insurers. He shows that a no-claim bonus and an experience-rated bonus even more, dampens the demand for ambulatory care. The experience-rated bonus implies that insured received a bonus of two monthly premiums in the first year with no claims, three monthly premiums in the second year, and four monthly premiums in the third consecutive year with no claims. Note, however, that with an ex-post differentiation, the premium is only differentiated to the number of previous consecutive years insured have opted for a voluntary deductible. The amount of claims is of no importance to the premium in the next year the insured opts for a voluntary deductible.

2 Only in the Health Insurance Marketplaces in the US (formerly known as the Health Insurance Exchanges) premiums may be conditioned on age (1:3), smoking (1:1.5), family size and geography, but not on other risk characteristics.

3 Note that the voluntary deductible in Germany holds for 3 years and insured can only adjust their voluntary deductible after these 3 years.

4 Note that due to the requirements of community-rating and open enrolment, insurers in regulated markets are, compared to insurers in free markets, unable to risk-rate the premium (rebate) or to deny insured to reduce the deductible level to counteract adverse selection.

5 In the corresponding amendment, this was originally formulated as: “the premium rebate may depend on the duration of the health insurance policy” [15], which is actually an ex-ante differentiation of the premium rebate.

6 In this paper, we solely focus on the insurance claims and disregard the insurers’ loading fee (e.g., overhead costs and administration costs), since in our data (“Data”) we have no information on the loading fee and since the loading fee only constitutes a small part of the premium.

7 In our dataset, few insured (0.9 %) opted for a voluntary deductible in 2011. Note that it is impossible to perform the empirical analyses with the insured who actually opted for a voluntary deductible since we only have data from one Dutch insurer. This means that we cannot follow insured who switch insurers. Research shows that especially young and healthy insured switch insurers [14, 23, 26]. These are also the insured who are most likely to opt for a voluntary deductible (e.g., [8, 11, 20, 31, 41]). Consequently, our results could become biased if we would perform the analyses with the insured who actually opted for a voluntary deductible in the dataset. To overcome this problem, we perform a simulation study. Prior to the simulation, we have corrected the healthcare expenses of the insured in the dataset who opted for a voluntary deductible in any year. For each of these insured in each of the years for the five different Dutch voluntary deductible levels (i.e., €100, €200, €300, €400, or €500), we increased their healthcare expenses with a percentage based upon extensive research by Keeler et al. [18] (see also [2, 3]), to correct for the moral hazard reduction that results from the voluntary deductible. Note that in this case we apply the results of the study by Keeler et al. [18] since this enables us to apply different percentages for different deductible levels in different years. For the moral hazard reduction in the rest of the paper, we apply the results by Trottmann et al. [30] since these results are more recent.

8 E.g., for the rank of the insured in year *t* − 1, this implies that the dependent variable regards the total healthcare expenses under basic insurance in year *t* − 1 and the independent variables regard an age and gender interaction, classification into a PCG and/or DCG in year *t* − 1 (based upon information from year *t* − 2), degree of urbanization in the residential area, ethnicity, and past total healthcare expenses in year *t* − 2 classified into vigintiles.

9 The average healthcare expenses of €1894 in the data regard the expenses without any cost sharing arrangements (i.e., the insurance claims, out-of-pocket expenses and moral hazard reduction as shown in Fig. 1). The average expenses are equal for all years due to the correction for inflation to the year 2007.

10 Note that, compared to Eq. (2b), the moral hazard reduction and out-of-pocket expenses in Eq. (2a) are omitted because insured without a voluntary deductible do not have any moral hazard reduction and out-of-pocket expenses resulting from a deductible.

11
*x* indicates the percentage in which risk equalization is able to equalize the predicted differences in healthcare expenses between low-risk and high-risk individuals caused by differences in health.

13 Note that the Swiss government has capped the offered premium rebate at 70 % of the voluntary deductible level and that all Swiss insurers offer this premium rebate. Due to a relatively poor risk equalization scheme (and therefore a substantial adverse selection component), it may be that if the Swiss government did not oblige this cap, premium rebates would be much higher.

## References

[1] Akerlof GA (1970). The market for ‘lemons’; quality uncertainty and the market mechanism. Q. J. Econ..

[2] Bakker, F.M.: Effecten van eigen betalingen op premies voor ziektekostenverzekeringen. Ph.D. Dissertation, Erasmus University Rotterdam, Rotterdam (1997).** (in Dutch)**

[3] Bakker FM, van Vliet RCJA, van de Ven WPMM (2000). Deductibles in health insurance: can the actuarially fair premium reduction exceed the deductible?. Health Policy.

[4] Buchner F, Goepffarth D, Wasem J (2013). The new risk adjustment formula in Germany: implementation and first experiences. Health Policy.

[5] Bundesamt für Gesundheit (2013). Statistik der obligatorischen Krankenversicherung 2013 (Statistics of the mandatory health insurance 2013).

[6] Cave J, Scheffler RM, Rossiter LF (1985). Equilibrium in insurance market with asymmetric information and adverse selection. Advances in health economics and health services research.

[7] Cummins JD, Smith BD, Vance N Van, Derhei J (1982). Risk classification in life insurance—Huebner international series on risk, insurance and economic security.

[8] Cutler, D.M., Zeckhauser, R.J.: The anatomy of health insurance. In: Culyer, A.J., Newhouse, J.P. (eds.) Handbook of Health Economics, vol. 1, pp. 563–643. Elsevier, Amsterdam (2000). doi:10.1016/S1574-0064(00)80170-5

[9] Cutler DM, Zeckhauser RJ (1998). Adverse selection in health insurance. Forum Health Econ. Policy.

[10] Folland S, Goodman AC, Stano M (2010). The economics of health and health care.

[11] Gabel, J.R., Lo Sasso, A.T., Rice, T.: Consumer-driven health plans: are they more than talk now? Health Aff. **22**(1), W395–W407 (2002) 10.1377/hlthaff.w2.395 12703601

[12] Gerfin M, Schellhorn M (2006). Nonparametric bounds on the effect of deductibles in health care insurance on doctor visits—Swiss evidence. Health Econ..

[13] Gerfin M, Kaiser B, Schmid C (2015). Healthcare demand in the presence of discrete price changes. Health Econ..

[14] Hendriks M, de Jong JD, Den Brink-Muinen V, Groenewegen PP (2009). The intention to switch health insurer and actual switching behavior: are there differences between groups of people?. Health Expect..

[15] House of Representatives: Amendement van het lid Omtzigt. Den Haag: The House of Representatives of the Dutch Parliament 29763, nr. 39 (2004)** (in Dutch)**

[16] iBMG: WOR 711 Onderzoek risicoverevening 2015: berekening normbedragen. Rotterdam: iBMG, Erasmus University Rotterdam. (2015)** (in Dutch)**

[17] Kautter J, Pope GC, Ingber M, Freeman S, Patterson L, Cohen M, Keenan P (2014). The HHS-HCC risk adjustment model for individual and small group markets under the Affordable Care Act. Med. Med. Res. Rev..

[18] Keeler, E.B., Buchanan, J.L., Rolph, J.E., Hanley, J.M., Reboussin, D.M.: The demand for episodes of medical treatment in the Health Insurance Experiment. RAND Report R3454-HHS, Santa Monica, CA (1988)

[19] Manning, W.G., Marquis, M.S.: Health Insurance: the trade-off between risk pooling and moral hazard. RAND, R-3729-NCHSR. (1989). doi:10.1016/S0167-6296(96)00497-3

[20] Marquis MS (1992). Adverse selection with a multiple choice among health insurance plans: a simulation analysis. J. Health Econ..

[21] Marquis, M.S., Holmer, M.R.: Choice under uncertainty and the demand for health insurance. RAND, N-2516-HHS (1986)

[22] Marquis, M.S., Phelps, C.E.: Price elasticity and adverse selection in the demand for supplementary health insurance. Econ. Inq. **25**(2) (1987). doi:10.1111/j.1465-7295.1987.tb00741.x 10.1111/j.1465-7295.1987.tb00741.x 10281607

[23] Mosca I, Schut-Welkzijn A (2008). Choice determinants of the mobility in the Dutch health insurance market. Eur. J. Health Econ.

[24] Neudeck W, Podczeck K (1996). Adverse selection and regulation in health insurance markets. J. Health Econ..

[25] Pauly MV (1986). Taxation, health insurance, and market failure in the medical economy. J. Econ. Lit..

[26] Reitsma-van Rooijen M, de Jong JD, Rijken M (2011). Regulated competition in health care: switching and barriers to switching in the Dutch health insurance system. BMC Health Serv. Res..

[27] Rothschild, M., Stiglitz, J.: Equilibrium in competitive insurance markets: an essay on the economics of imperfect information. Q. J. Econ. 629–649 (1976). doi:10.1007/978-94-015-7957-5_18

[28] Shmueli A, Stam P, Wasem J, Trottmann M (2015). Managed care in four managed competition OECD systems. Health Policy.

[29] Steinbusch PJM, Oostenbrink JB, Zuurbier JJ, Schaepkens FJM (2007). The risk of upcoding in casemix systems: a comparative study. Health Policy.

[30] Trottmann M, Zweifel P, Beck K (2012). Supply-side and demand-side cost sharing in deregulated social health insurance: which is more effective?. J. Health Econ..

[31] Van de Ven WPMM, van Praag BMS (1981). The demand for voluntary deductibles in private health insurance: a probit model with sample selection. J. Econ..

[32] Van de Ven WPMM, van Vliet RCJA (1995). Consumer information surplus and adverse selection in competitive health insurance markets: an empirical study. J. Health Econ..

[33] Van Kleef RC, van de Ven WPMM, van Vliet RCJA (2006). A voluntary deductible in social health insurance with risk equalization: “community-rated or risk-rated premium rebate?”. J. Risk Insur..

[34] Van Kleef RC, Beck K, van de Ven WPMM, van Vliet RCJA (2007). Does risk equalization reduce the viability of voluntary deductibles?. Int. J. Health Care Financ. Econ..

[35] Van Kleef RC, Beck K, Van de Ven WPMM, Van Vliet RCJA (2008). Risk equalization and voluntary deductibles: a complex interaction. J. Health Econ..

[36] Van Kleef, R.C., Beck, K., Buchner, F.: How self-selection affects risk equalization: the example of voluntary deductibles. In: Van Kleef, R.C. (Ed.) Voluntary deductibles and risk equalization: a complex interaction. Ph.D. Dissertation, Erasmus University Rotterdam, Rotterdam (2008b)

[37] Van Kleef RC, van Vliet RCJA, van de Ven WPMM (2013). Risk equalization in the Netherlands: an empirical evaluation. Expert Rev. Pharmacoecon. Outcomes Res..

[38] Van Winssen KPM, van Kleef RC, van de Ven WPMM (2015). Potential determinants of deductible uptake in health insurance: how to increase the uptake in the Netherlands?. Eur. J. Health Econ..

[39] Vektis.: Zorgthermometer: verzekerden in beeld 2015. Vektis, Zeist (2015)** (in Dutch)**

[40] Wilson C (1977). A model of insurance markets with incomplete information. J. Econ. Theory.

[41] Wolfe JR, Goddeeris JH (1991). Adverse selection, moral hazard, and wealth effects in the Medigap insurance market. J. Health Econ..

[42] Zweifel P (1987). Bonus systems in health insurance: a microeconomic analysis. Health Policy.

[43] Zweifel, P., Manning, W.: Moral hazard and consumer incentives in health care. In: Culyer, A.J., Newhouse, J.P. (eds.) Handbook of Health Economics, vol. 1, pp. 409–459. Elsevier, Amsterdam (2000). doi:10.1016/S1574-0064(00)80167-5

[44] Zweifel P, Breyer F, Kifmann M (2009). Health Economics.

